# Integration of Photon-Counting CT into the Surgical Workflow of Complex Maxillofacial Reconstruction: A Pilot Feasibility Study

**DOI:** 10.3390/diagnostics16060876

**Published:** 2026-03-16

**Authors:** Ioanna Kalaitsidou, Matias Maissen, Florian Dammann, Christian Schedeit, Daniel Jan Toneatti, Benoît Schaller

**Affiliations:** 1 Department of Cranio-Maxillofacial Surgery, Inselspital, Bern University Hospital, CH-3010 Berne, Switzerland; matias.maissen@students.unibe.ch (M.M.); christian.schedeit@insel.ch (C.S.); daniel.toneatti@insel.ch (D.J.T.); benoit.schaller@insel.ch (B.S.); 2 Department of Radiology, Inselspital, Bern University Hospital, CH-3010 Berne, Switzerland; florian.dammann@insel.ch

**Keywords:** photon-counting computed tomography, oral and maxillofacial surgery, reconstructive surgical procedures, surgical planning, computer-assisted surgery, free tissue flaps, head and neck neoplasms

## Abstract

**Background/Objectives:** Virtual surgical planning (VSP) and CAD/CAM technologies have revolutionized complex maxillofacial reconstruction. While high-resolution imaging is critical for these workflows, the specific clinical impact of photon-counting computed tomography (PCCT) remains to be fully established. This prospective pilot study evaluates the feasibility and clinical utility of integrating PCCT into the preoperative planning and surgical workflow of complex maxillofacial reconstructive cases. **Methods:** This feasibility study included ten patients requiring complex maxillofacial reconstruction with microvascular free flaps. All underwent preoperative imaging with photon-counting CT. Primary endpoints included clinical assessment of osseous invasion, reliability of donor-site vascular mapping from a single acquisition, and compatibility of PCCT datasets with VSP/CAD-CAM platforms. Secondary endpoints included resection margin status, flap survival, and short-term oncologic outcomes. **Results:** PCCT provided high-resolution visualization of cortical and medullary bone, enabling detailed assessment of tumor-related osseous involvement. In selected cases, findings supported refinement of resection planning when prior imaging had been inconclusive. Spectral reconstructions reduced metal artifacts and facilitated precise segmentation for multi-segment osteotomies. Donor-site vascular anatomy was successfully evaluated within the same scan, supporting operative planning without additional imaging. PCCT datasets were fully compatible with the virtual surgical planning (VSP) software used in this study (CMX Portal, version 2.6.1158, Medartis AG, Basel, Switzerland; or ProPlan CMF, version 5.7.8.025, Materialise NV, Leuven, Belgium) in all cases (100%). Reconstruction was completed successfully in all patients, with 100% flap survival and R0 margins in all malignant cases. No technical failures occurred during imaging transfer or CAD/CAM fabrication. **Conclusions:** The integration of PCCT into the surgical workflow proved technically feasible and clinically impactful. This pilot data supports its potential to enhance surgical precision and preoperative planning in complex jaw reconstruction.

## 1. Introduction

Osseous reconstruction in the facial region is a cornerstone of modern oral and maxillofacial surgery, aiming to restore both function and aesthetic integrity following segmental resections caused by a broad range of pathologies [1]. These include malignant neoplasms, benign tumors, traumatic injuries, and complications such as osteoradionecrosis (ORN) [1,2]. In these complex cases, successful rehabilitation depends not only on the surgical technique but also on precise preoperative imaging and planning [2]. The choice of reconstructive method is determined by multiple factors, including the anatomical complexity of the defect (bone, mucosa, muscle, skin), patient comorbidities, functional requirements, and aesthetic expectations [3]. The fibula free flap (FFF) is widely considered a primary reconstruction option due to its robust bone stock and reliable vascular pedicle. However, alternative flaps such as the deep circumflex iliac artery (DCIA) flap, scapular flaps, the anterolateral thigh (ALT) flap, radial forearm flap, and superficial circumflex iliac perforator (SCIP) flap have been employed in selected cases [4,5,6].

Contemporary surgical approaches include osseous microvascular free flaps, with or without simultaneous dental implant placement, or the use of pre-bent reconstruction plates combined with soft tissue flaps. Each technique requires a comprehensive understanding of the defect’s three-dimensional anatomy, vascular supply, and the interplay between hard and soft tissue structures [7,8,9]. This demands meticulous diagnostic radiological procedures to ensure effective treatment. The tumor staging process currently relies on biopsy, panendoscopy, and modern imaging technology, such as CT, MRI, and PET-CT scans [9,10].

Computed tomography (CT) has demonstrated a sensitivity of up to 95% for osseous infiltration, including cortical erosion and bone marrow space debilitation. However, it is significantly limited by its susceptibility to metallic artifacts, particularly in patients with dental restorations or implants, which can obscure diagnostic clarity and compromise surgical planning [11,12,13]. This limitation has prompted the exploration of Photon-Counting CT (PCCT) as a next-generation imaging modality [14]. PCCT utilizes photon-counting detectors (PCDs) that directly convert incoming X-ray photons into electrical signals. Unlike energy-integrating detectors (EID) used in conventional CT, PCDs offer higher spatial resolution, reduced image noise, enhanced soft tissue contrast, and significantly improved metal artifact reduction (MAR). These features are particularly advantageous in the complex anatomical landscape of the maxillofacial region [14].

The integration of computer-aided design and manufacturing (CAD/CAM) into maxillofacial surgery has further revolutionized preoperative planning. Patient-specific cutting guides and implants enhance surgical precision and reduce intraoperative time. However, the full potential of these technologies hinges on the quality of imaging input, and PCCT, with its detailed spectral and anatomical outputs, offers promising synergy with CAD/CAM workflows [15,16,17,18].

Despite the growing clinical interest in photon-counting computed tomography (PCCT), its specific role within the domain of oral and maxillofacial surgery remains largely undefined. Prior studies have focused predominantly on neurological, cardiovascular, or orthopedic applications, with limited evidence regarding its integration into reconstructive surgical workflows. Moreover, the potential of PCCT to enhance virtual surgical planning (VSP), reduce imaging artifacts, and consolidate vascular assessment into a single scan has not yet been systematically evaluated in the context of complex maxillofacial reconstruction.

This pilot study addresses that gap by investigating the clinical feasibility of incorporating PCCT into the full surgical workflow of patients requiring microvascular free flap reconstruction of the jaws. Specifically, we evaluate its diagnostic precision for osseous invasion, its utility in donor site vascular mapping, and its compatibility with CAD/CAM-based planning platforms. To our knowledge, this is the first study to demonstrate a fully integrated PCCT-to-VSP pathway in real-world oncologic cases requiring complex osseous reconstruction, underscoring its feasibility and potential value within digital reconstructive maxillofacial workflows.

## 2. Materials and Methods

### 2.1. Study Design and Patient Selection

This pilot feasibility study was conducted at the Department of Cranio-Maxillofacial Surgery in collaboration with the Department of Radiology at the University Hospital of Bern (Inselspital). A consecutive cohort of ten oncologic patients (*n* = 10) requiring complex maxillofacial reconstruction was prospectively enrolled. Inclusion criteria comprised patients scheduled for microvascular free flap reconstruction due to either malignant neoplasm, for whom high-precision virtual surgical planning (VSP) was clinically indicated. Histopathologic diagnoses included primary squamous cell carcinoma (SCC), recurrent or persistent SCC, and one malignancy arising from minor salivary glands. All patients required high-precision preoperative imaging to define tumor-related osseous involvement and to enable digital workflow integration.

The study was approved by the local institutional review board (Kantonale Ethikkommission Bern; protocol number: 2025-00872) and conducted in accordance with the Declaration of Helsinki. Informed consent was obtained from all subjects involved in the study.

The limited sample size reflects the exploratory nature of this feasibility study, consistent with early-phase imaging protocol validation frameworks. As the primary objective was to evaluate the clinical integration and technical usability of photon-counting CT (PCCT) in a surgical workflow, no formal sample size calculation was performed. The findings aim to generate foundational data and inform the design of future prospective studies with powered statistical analyses.

### 2.2. Study Endpoints

The primary objective of this pilot study was to evaluate the technical feasibility of integrating photon-counting CT (PCCT) into a fully digital reconstructive workflow. Primary endpoints included the following:

(1) Successful transfer of PCCT data into the CAD/CAM software platforms (CMX Portal, version 2.6.1158, Medartis AG, Basel, Switzerland; or ProPlan CMF, version 5.7.8.025, Materialise NV, Leuven, Belgium);
(2) Adequacy of tumor-related osseous delineation for surgical margin planning;
(3) Reliability of donor-site vascular assessment within the same imaging session.

Secondary endpoints included intraoperative guide fit, flap survival, resection margin status (R0/R1), and short-term oncologic outcomes.

### 2.3. Imaging Protocol (Photon-Counting CT)

All patients underwent preoperative imaging using a first-generation dual-source PCCT scanner (NAEOTOM Alpha; Siemens Healthineers, Forchheim, Germany). The scanning parameters were standardized to ensure high-fidelity data for CAD/CAM integration. All scans were performed at a tube voltage of 120 kV using the Quantum Plus technology. To optimize image quality and reduce radiation dose, an automated exposure control (CARE Dose4D) was utilized.

The PCCT detector utilized cadmium telluride (CdTe) crystals, enabling direct conversion of X-ray photons into electrical signals with a spatial resolution of up to 0.2 mm. For cases requiring vascular mapping of the donor site (e.g., fibula free flap), a biphasic contrast-enhanced protocol was applied using iopromide (Ultravist 370; Bayer Healthcare, Berlin, Germany), followed by a saline bolus (Table 1). Donor-site vascular anatomy was evaluated during the same PCCT session, allowing assessment of arterial patency and anatomical variations relevant for flap harvest planning. All PCCT datasets were reconstructed and exported in DICOM format to ensure full compatibility with segmentation software used for virtual surgical planning (VSP) and CAD/CAM fabrication.

### 2.4. Image Reconstruction and Metal Artifact Reduction (MAR)

Image data were reconstructed using specialized kernels (e.g., Br64 for bone and Br40 for soft tissue) to maximize the contrast-to-noise ratio. A slice thickness of 0.2 mm to 0.4 mm with an increment of 0.1–0.2 mm was used for all bony reconstructions. To address the significant diagnostic challenge posed by dental implants and osteosynthesis material, spectral thermal maps and iterative reconstruction algorithms (Quantum Iterative Reconstruction, QIR) were employed. Specifically, virtual monoenergetic images (VMI) at high energy levels (100–140 keV) were utilized to minimize metal artifacts and enhance the visibility of the bone-implant interface.

### 2.5. Virtual Surgical Planning (VSP) and CAD/CAM Integration

The DICOM datasets from the PCCT were imported into dedicated virtual surgical planning software (CMX Portal, version 2.6.1158, Medartis AG, Basel, Switzerland; or ProPlan CMF, version 5.7.8.025, Materialise NV, Leuven, Belgium). The segmentation of the maxillofacial skeleton and the donor bone was performed based on the high-resolution PCCT slices. Image segmentation was performed using semi-automated thresholding tools within the virtual planning software, followed by manual refinement by a single experienced maxillofacial surgeon. No inter-observer variability testing was performed due to the exploratory nature of the pilot study. All segmentations were validated intraoperatively based on real-time anatomical correlation. The feasibility of the workflow was evaluated based on the following:

- Diagnostic Precision: The ability to delineate tumor margins and medullary invasion.
- Workflow Integration: The seamless transfer of PCCT datasets into the CAD/CAM planning software (CMX Portal, version 2.6.1158, Medartis AG, Basel, Switzerland; or ProPlan CMF, version 5.7.8.025, Materialise NV, Leuven, Belgium) for the fabrication of 3D-printed cutting guides and patient-specific implants (PSI).
- Intraoperative Correlation: The correlation between the PCCT-based virtual plan and the actual surgical findings.

Quantitative image quality metrics such as contrast-to-noise ratio (CNR) or signal-to-noise ratio (SNR) were not computed, as the primary study aim was clinical feasibility and integration. However, subjective image quality and artifact suppression were assessed during VSP and surgical planning and deemed sufficient for precise resection margin and vessel delineation in all cases.

Overall, this methodological design aimed to simulate a real-world clinical workflow using PCCT for preoperative planning in complex reconstructions, focusing on technical feasibility, data transfer fidelity, and practical surgical applicability.

### 2.6. Surgical Procedure

All patients underwent segmental jaw resection followed by immediate microvascular free flap reconstruction. Flap selection and design were based on virtual plans and vascular assessment from the PCCT angiographic datasets. Intraoperative execution was guided by prefabricated cutting guides and customized implants.

### 2.7. Statistical Analysis

Given the pilot design and limited sample size (*n* = 10), statistical analysis was primarily descriptive. Continuous variables are presented as mean and range, and categorical variables as counts and percentages. Primary and secondary endpoints were analyzed descriptively. No inferential statistical comparisons were performed, as the study was not powered for hypothesis testing.

## 3. Results

A total of ten consecutive oncologic patients (7 male, 3 females; mean age 63.1 years, range 49–77) underwent complex maxillofacial reconstruction following preoperative evaluation with photon-counting CT (PCCT). Histopathological diagnoses comprised primary squamous cell carcinoma (SCC) (*n* = 7), recurrent or persistent SCC (*n* = 2), and one malignancy arising from minor salivary glands (mucoepidermoid carcinoma) (*n* = 1). All patients successfully completed the full PCCT-to-CAD/CAM workflow, including imaging, segmentation, virtual surgical planning (VSP), and intraoperative execution using patient-specific guides and implants.

### 3.1. Diagnostic Performance and Surgical Decision-Making

**Figure 1:**
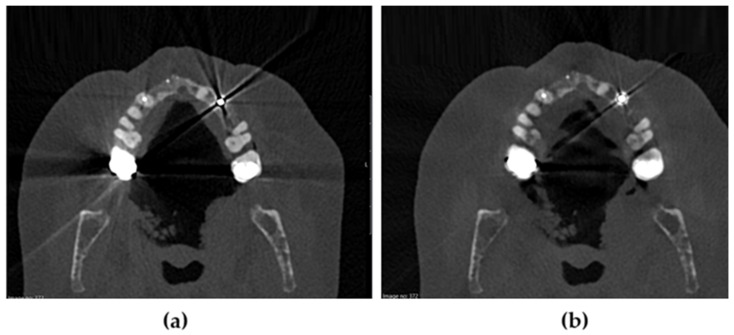
Effect  of iterative metal artifact reduction (IMAR) on maxillofacial photon-counting CT (PCCT). Axial ultra-high-resolution (0.2 mm kernel) PCCT images of the maxillofacial region demonstrating the impact of metal artifact reduction. (**a**) Image reconstructed without IMAR, showing pronounced streak artifacts originating from dental metallic restorations, partially obscuring adjacent cortical bone and surrounding soft tissues. (**b**) Image reconstructed with IMAR, demonstrating substantial reduction in streak artifacts and improved delineation of mandibular cortices and adjacent anatomical structures.

PCCT imaging provided high-resolution visualization of cortical and medullary bone structures in all ten oncologic patients, with minimal metal artifact interference. Representative ultra-high-resolution axial images demonstrating the effect of iterative metal artifact reduction (IMAR) are shown in Figure 1. Image quality was consistently sufficient for accurate assessment of tumor-related osseous involvement and integration into virtual surgical planning (VSP).

**Figure 2:**
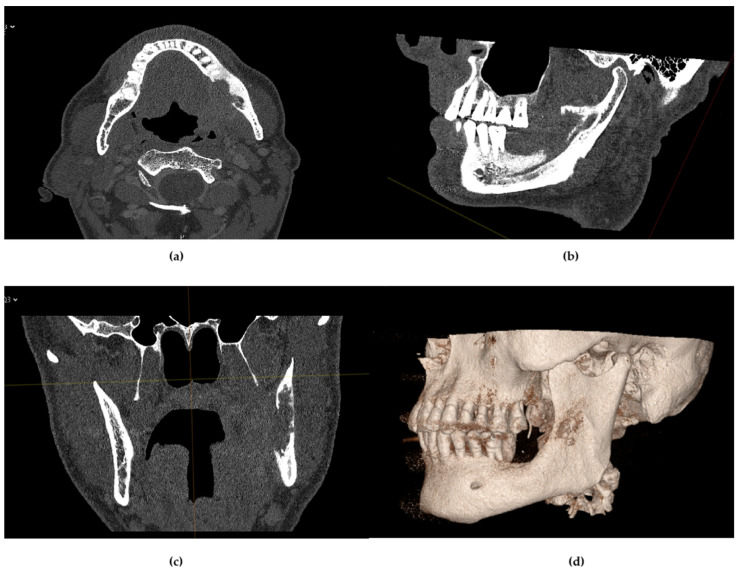
Multiplanar PCCT evaluation of mandibular tumor infiltration. Axial (**a**), sagittal (**b**), and coronal (**c**) reconstructions demonstrate cortical erosion and medullary extension of the lesion. Three-dimensional volume rendering (**d**) illustrates defect morphology and supports preoperative virtual surgical planning.

In two of ten cases (20%), PCCT revealed previously underappreciated medullary bone invasion that was not clearly demonstrated on prior imaging modalities. In both cases (Cases 1 and 9), this led to oncologic upstaging from cT3 to cT4a and modification of the surgical plan toward wider segmental mandibulectomy. In three cases (Cases 2, 8 and 10), PCCT provided clear delineation of residual maxillary bone margins in a previously operated field, enabling confident definition of oncologic resection limits. Although reconstruction was deferred to a secondary stage, the imaging supported a conservative resection strategy without compromising oncologic safety. In one additional case (Case 4, 10%), contrast-enhanced PCCT identified posterior tibial artery occlusion during donor-site evaluation, prompting selection of the contralateral lower extremity for fibula harvest. In Case 10, given the limited vertical bone requirement and favorable defect morphology, a SCIP flap was considered reconstructively adequate. In the context of the patient’s preference to avoid potential lower limb donor-site morbidity, this option was selected following multidisciplinary evaluation and shared decision-making. Across the remaining cases, PCCT enabled precise delineation of cortical and medullary boundaries, supporting confident definition of safe resection margins and accurate design of patient-specific implants and cutting guides. No intraoperative discrepancies were observed between PCCT-based planning and surgical findings (Figure 2).

Demographic data, surgical details, and clinical outcomes are summarized in Table 2.

### 3.2. Integration into Virtual Planning Workflow

Segmentation and virtual planning were successfully completed in all cases using PCCT datasets. No compatibility issues were encountered during DICOM transfer to the virtual surgical planning (VSP) platforms (either CMX Portal Medartis or ProPlan CMF, depending on the individual case). In four patients, multi-segment osteotomies were required; in all instances, the patient-specific cutting guides demonstrated excellent intraoperative fit without the need for trimming or modification. Although segmentation and planning times were not systematically recorded, the overall imaging-to-surgery timeline remained within standard institutional protocols. No case required repeat imaging due to inadequate dataset quality. These findings confirm the technical feasibility of integrating PCCT into a fully digital reconstructive workflow.

### 3.3. Vascular Assessment and Donor Site Planning

**Figure 3:**
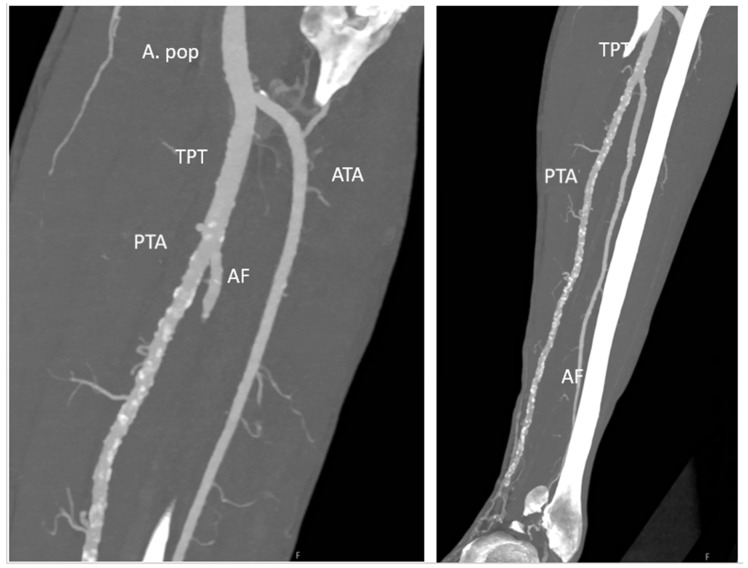
Lower limb vascular assessment using photon-counting CT (PCCT). Coronal maximum intensity projection (MIP, 10 mm) of the left lower extremity acquired with PCCT. The popliteal artery (A. poplitea), anterior tibial artery (ATA), posterior tibial artery (PTA), tibioperoneal trunk (TPT), and fibular artery (AF) are visualized, enabling preoperative donor-site vascular evaluation within a single imaging acquisition.

Contrast-enhanced PCCT angiographic reconstructions reliably visualized the arterial anatomy of the lower extremities in all ten cases. This obviated the need for separate CT angiography or Doppler ultrasound. In one patient (Case 4, 10%), a previously unrecognized posterior tibial artery occlusion was identified, prompting a shift in fibula harvest location to preserve vascular supply. These findings confirmed that high-resolution vascular mapping can be achieved through a single PCCT acquisition without compromise in image quality or diagnostic confidence (Figure 3).

### 3.4. Surgical and Oncologic Outcomes

All ten patients underwent successful reconstruction using microvascular free flaps. The fibula free flap (FFF) was used in nine cases, while one patient underwent reconstruction using an osteocutaneous SCIP flap. There were no flap losses (100% survival), and no major perioperative complications were observed. Histopathologically confirmed R0 resection margins were achieved in all cases (10/10). All patients recovered uneventfully and were discharged within the expected postoperative timeframe.

**Figure 4:**
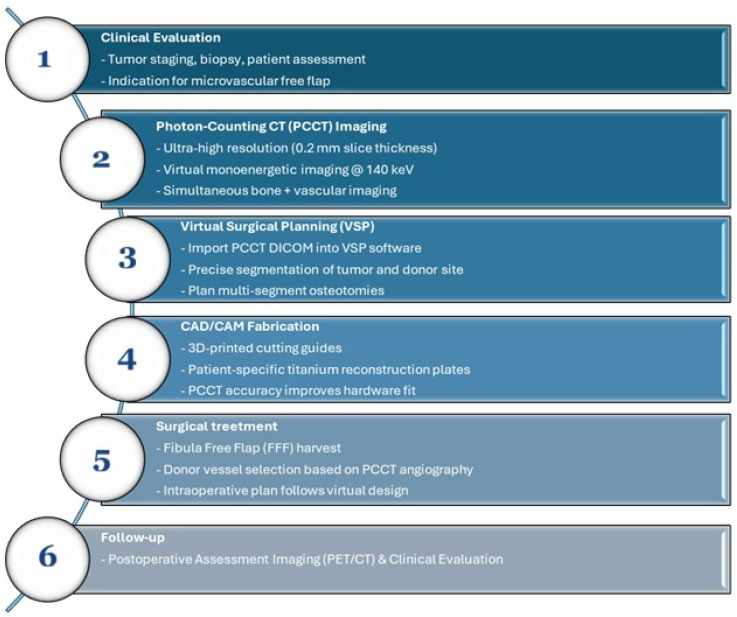
PCCT-based virtual surgical planning workflow.

All patients were followed for a minimum of 12 months postoperatively (range: 12–16 months), with scheduled clinical and imaging assessments at regular intervals. No cases of local recurrence, donor site morbidity, or flap-related complications were observed during this period. Surveillance imaging (MRI or PET/CT, as indicated) confirmed disease-free status at final assessment in all cases. In all cases, implant-supported prosthetic rehabilitation is planned as the definitive reconstructive goal. Where immediate implant placement was not feasible, staged rehabilitation is scheduled following appropriate healing and osseointegration assessment (Figure 4).

### 3.5. Observations on Workflow Feasibility and Subjective Image Quality

The integration of PCCT datasets into the virtual surgical planning workflow was technically feasible in all ten cases. DICOM export, segmentation, and CAD/CAM processing were completed without technical limitations or need for repeat imaging.

Subjective assessment of image quality by both radiologists and surgeons indicated consistently high diagnostic confidence with regard to cortical delineation, medullary extension, and segmentation suitability. Artifact suppression in the presence of dental restorations or previous reconstruction plates was considered adequate for surgical planning purposes.

Although no formal image quality scoring system was applied, the datasets were deemed sufficient for clinical decision-making and workflow integration in all cases.

While this pilot cohort yielded uniformly favorable outcomes, the limited sample size (*n* = 10) and absence of direct comparative imaging (e.g., conventional CT, standalone CTA) preclude definitive conclusions on diagnostic superiority. Nonetheless, these findings support the feasibility of consolidating oncologic and vascular imaging into a single PCCT acquisition, potentially streamlining preoperative planning in complex maxillofacial reconstruction (Table 3).

## 4. Discussion

Photon-counting computed tomography (PCCT) represents a fundamental technological evolution in medical imaging [19] and, as demonstrated in this pilot study, offers potentially clinically relevant advantages for complex maxillofacial reconstruction. Unlike energy-integrating detector (EID) systems that rely on scintillation-based signal conversion, PCCT employs cadmium telluride (CdTe) semiconductor detectors that directly convert X-ray photons into electrical signals, thereby reducing electronic noise and enabling inherent spectral imaging capabilities [20,21,22]. In the present cohort, these technical advantages translated into high diagnostic confidence and robust compatibility with digital surgical planning.

The diagnostic impact of PCCT was most evident in oncologic cases, where ultra-high spatial resolution enabled detection of subtle osseous changes that were inconclusive on conventional imaging. In Case 1, PCCT revealed medullary mandibular invasion that was occult on MRI and PET/CT, resulting in immediate disease upstaging from cT3 to cT4a and a change in surgical strategy from marginal to segmental mandibulectomy. A similar effect was observed in Case 9, in which PCCT-supported delineation of medullary extension contributed to oncologic upstaging and resection refinement. These findings underscore the role of PCCT as a problem-solving modality when standard imaging techniques yield equivocal results. Chang et al. noted that ultra-high-resolution (UHR) protocols can increase image noise; however, the application of deep learning-based denoising using convolutional neural networks (CNNs) enables preservation of spatial detail down to 0.11 mm [23], which likely contributed to the high diagnostic confidence observed in our series.

Beyond oncologic staging, PCCT demonstrated significant value in the evaluation of compromised bone quality and internal trabecular structure [24], which may be particularly relevant in previously treated or irradiated oncologic fields where differentiation of altered bone from viable structures can be challenging. In the present cohort, PCCT supported clear visualization of cortical integrity and trabecular architecture in complex surgical fields, facilitating definition of resection limits and reconstruction planning. These observations align with emerging evidence from bone imaging research. Hermans et al. demonstrated that PCCT outperforms multidetector CT (MDCT) in 13 of 14 anatomical landmarks in temporal bone imaging, offering superior resolution of fine osseous structures [25]. Furthermore, cadaveric studies by Quintiens et al. and Shi et al. have shown strong correlation between PCCT, micro-CT, and high-resolution peripheral quantitative CT (HR-pQCT) in quantifying bone porosity and trabecular integrity [26,27]. Our results suggest that this “micro-CT-like” imaging capability is clinically transferable to the maxillofacial skeleton and can supporting precise surgical planning in selected complex oncologic reconstructions.

Metal artifact reduction remains a persistent challenge in maxillofacial imaging, particularly in patients with prior dental hardware or reconstructive implants [28]. In our series, spectral PCCT with virtual monoenergetic imaging (VMI) at 140 keV achieved effective suppression of streak artifacts, enabling accurate segmentation of the bone–implant interface in all cases. This clinical observation validates experimental findings by Pallasch et al., who reported superior diagnostic quality when using iterative metal artifact reduction (iMAR) combined with high-energy VMI [29]. Sawall et al. also demonstrated that PCCT provides 31–37% higher contrast-to-noise ratios (CNRD) for enamel–dentin and dentin–bone interfaces compared to CBCT, and at lower radiation doses [30]. Similarly, Vanden Broeke et al. confirmed PCCT’s superiority over CBCT in visualizing soft and hard tissues adjacent to metallic structures [28]. These findings support the use of PCCT for accurate patient-specific implant design, even in imaging environments previously considered suboptimal.

A key strength of our protocol is the seamless integration of PCCT data into the virtual surgical planning (VSP) workflow. In all cases, PCCT datasets were successfully imported into VSP software without format conversion or technical delays. Segmentation was performed using semi-automated tools followed by manual refinement by a single experienced surgeon, typically within 30–40 min. The excellent intraoperative fit of cutting guides mirrors findings from Klintström et al., whose preclinical study demonstrated that PCCT offers superior bone edge delineation and soft tissue visualization compared to intraoperative CBCT [31]. Additionally, PCCT enabled simultaneous high-resolution vascular imaging, eliminating the need for separate CT angiography. In one case, donor-site planning was directly altered after PCCT angiography revealed an unsuspected vascular occlusion. These results align with reports by Rak et al. and Doyle et al., who showed PCCT achieves diagnostic performance comparable to micro-CT in fine otologic and neurovascular structures [32,33]. Importantly, this “one-stop” approach may also reduce overall radiation burden. Several studies have reported PCCT enables dose reductions of up to 67–80% compared to traditional protocols while maintaining image quality [34,35], a particularly important consideration in oncologic populations requiring serial follow-up imaging. In the present pilot cohort, formal dose comparison was not performed; therefore, potential dose advantages should be interpreted in the context of the cited literature rather than as a direct outcome of this study.

From a reconstructive perspective, microvascular osseous flaps remain central to segmental jaw reconstruction following oncologic resection [1,2,6]. The fibula free flap (FFF) is widely used due to its bone stock, pedicle length, and versatility [1,7,8]. However, flap choice is defect-dependent and center-specific, and alternative osseous flaps such as DCIA and scapular-based flaps remain established options in selected scenarios [2,3].

In this cohort, FFF was used in the majority of cases, while one patient underwent reconstruction with an osteocutaneous SCIP flap. In that case, the limited vertical bone requirement and favorable defect morphology rendered SCIP reconstructively adequate [5], and the choice was further supported by the patient’s preference to avoid potential lower limb donor-site morbidity following multidisciplinary evaluation and shared decision-making.

Soft-tissue flaps such as the anterolateral thigh (ALT) flap and radial forearm free flap (FRFF) serve different reconstructive purposes and may be used alone with reconstruction plates or in combination with osseous flaps when indicated, particularly in advanced oncologic settings [4,6]. Their role should therefore be interpreted separately from primary osseous free-flap reconstruction.

In one additional patient, a combined FFF and ALT flap was performed due to a large postoperative extraoral soft-tissue defect requiring additional external coverage. In this setting, the ALT flap was used to restore external contour and soft-tissue volume, while the fibula provided the osseous framework for mandibular reconstruction. Despite the encouraging results, this study has limitations that should be acknowledged. The sample size was limited (*n* = 10), with no control group or formal statistical analysis, limiting generalizability and preventing robust comparison with conventional modalities. All segmentations were performed by a single observer without inter-rater agreement testing, introducing possible bias. Moreover, quantitative metrics for image quality such as signal-to-noise ratio (SNR) or contrast-to-noise ratio (CNR) were not computed, as the study’s focus was on feasibility and integration. Subjective assessments, however, consistently deemed PCCT image quality sufficient for detailed surgical planning. From a systemic standpoint, PCCT faces real-world implementation barriers including high capital cost—estimated at three to four times that of conventional CT [20,36], and increased data volume that may tax PACS infrastructure and reporting workflows.

Future studies should explore these limitations in a controlled and comparative framework. Prospective, multicenter trials comparing PCCT with standard CT and MRI will be essential to determine its true diagnostic and cost-effectiveness value. Quantitative evaluation of segmentation time, interobserver reproducibility, margin accuracy, and radiation dose will help refine best practices. Furthermore, the potential for PCCT to perform material decomposition, a unique feature, may unlock new clinical applications, such as differentiating tumor from post-treatment inflammation. Integration with AI-driven segmentation and planning tools may also enhance efficiency, standardization, and accessibility.

## 5. Conclusions

Photon-counting computed tomography represents an emerging imaging technology with potential applications in oral and maxillofacial surgery. In this pilot cohort of ten oncologic patients undergoing complex jaw reconstruction, integration of PCCT into a fully digital workflow proved technically feasible and compatible with virtual surgical planning and CAD/CAM-based reconstruction. PCCT provided high-resolution visualization of osseous structures and enabled simultaneous vascular assessment within a single acquisition protocol. In selected cases, imaging findings contributed to refinement of surgical planning. However, given the limited sample size and absence of direct comparative imaging, no conclusions regarding diagnostic superiority can be drawn. These preliminary findings support further prospective, controlled studies to evaluate the diagnostic performance, workflow impact, radiation dose profile, and cost-effectiveness of PCCT in maxillofacial surgical planning.

## Figures and Tables

**Table 1:** CT acquisition parameters for photon-counting imaging.

Parameter	Value
Scanner	NAEOTOM Alpha (Siemens Healthineers, Forchheim, Germany)
Detector Type	Cadmium Telluride (CdTe) PCD
Tube Voltage	120 kV
Tube Current Modulation	CARE Dose4D (automated)
Slice Thickness	0.2–0.4 mm
Reconstruction Increment	0.1–0.2 mm
Kernels	Br64 (bone), Br40 (soft tissue)
Contrast Agent	Iopromide (Ultravist 370; 100 mL @ 4 mL/s)
Saline Bolus	30 mL post-contrast
Angiography Protocol	Biphasic (arterial + venous phase)

**Table 2:** Patient characteristics, surgical goals, and outcomes (Abbreviations: SCC: Squamous Cell Carcinoma; MEC: mucoepidermoid carcinoma; FFF: Fibula Free Flap; R0: Negative Margins; Lt: Left; Rt: Right).

Case	Age/Sex	Diagnosis & Site	Reconstruction (Flap)	Reconstruction Goal	Outcome	Specific PCCT Contribution
1	68/M	SCC, Floor of Mouth	FFF	Segmental Resection	R0/ Success	Detected medullary invasion (occult on MRI); Upstaged from cT3 to cT4a.
2	59/F	SCC, Maxilla	FFF	Brown IIb Maxillectomy	R0/ Success	High-resolution delineation of residual bone for PSI fit.
3	56/M	SCC, Retromolar	FFF	Hemimandibulectomy	R0/ Success	Defined cortical & medullary extension for safe margins.
4	67/M	Recurrent SCC, Mandible	FFF (2-seg)	Subtotal Mandibulectomy	R0/ Success	Detected vessel occlusion (Rt leg); Guided switch to Lt leg.
5	59/F	SCC, Floor of Mouth	FFF (2-seg) + ALT flap	Mandibulectomy	R0/ Success	Defined cortical & medullary extension for safe margins.
6	77/F	SCC, Floor of Mouth & Mandible	FFF (2-seg)	Mandibulectomy	R0/ Success	Defined cortical & medullary extension for safe margins.
7	65/M	Tumor persistence SCC, Mandible	FFF	Mandibulectomy	R0/ Success	High-resolution imaging enabled differentiation between postoperative changes and tumor persistence
8	68/M	SCC, Maxilla	FFF	Brown IIb Maxillectomy	R0/ Success	High-resolution delineation of residual bone for PSI fit.
9	49/M	Tumor persistence/progression of mucoepidermoid carcinoma (MEC), Floor of Mouth	FFF (2-seg)	Mandibulectomy	R0/ Success	Detected medullary invasion (occult on MRI); Upstaged from cT3 to cT4a.
10	63/M	SCC, Maxilla	Osteocutaneous SCIP flap	Brown IIb Maxillectomy	R0/ Success	High-resolution delineation of residual bone for PSI fit.

**Table 3:** Summary of imaging-to-surgery workflow and clinical outcomes.

Metric	Value/Observation
Number of patients	10
Indications	SCC (*n* = 9), MEC (*n* = 1)
Mean Age	63.1 years (range 49–77)
Segmentation Method	Semi-automated + manual refinement
Segmentation/Planning Software	CMX Portal Medartis, ProPlan CMF
Virtual Surgical Planning Compatibility	100% (no DICOM transfer failures)
Flap Type	FFF (*n* = 9), SCIP (*n* = 1)
Intraoperative Fit of Guides	Excellent; no modifications required
Resection Margins (oncologic cases)	R0 achieved in 10/10
Flap Survival	100%
Postoperative Complications	None
Follow-up Duration	12–16 months
Local Recurrence	None

## Data Availability

The data presented in this study are available on reasonable request from the corresponding author. The data are not publicly available due to patient confidentiality and institutional data protection regulations.

## Abbreviations

The following abbreviations are used in this manuscript:

CT	Computed Tomography
VSP	Virtual surgical planning
CAD/CAM	Computer-Aided Design and Computer-Aided Manufacturing
PCCT	Photon-Counting CT
ORN	Osteoradionecrosis
FFF	Fibula free flap
DCIA	Deep circumflex iliac artery
ALT	Anterolateral Thigh
SCIP	Superficial Circumflex Iliac Perforator
MRI	Magnetic Resonance Imaging
PET/CT	Positron Emission Tomography/Computed Tomography
PCD	Photon-Counting Detectors
EID	Energy Integrating Detectors
MAR	Metal artifact reduction
CdTe	Cadmium telluride
QIR	Quantum Iterative Reconstruction
VMI	Virtual monoenergetic images
DICOM	Digital Imaging and Communications in Medicine
PSI	Patient-specific implants
CNR	Contrast-To-Noise Ratio
SNR	Signal-to-noise ratio
OCSCC	Oral Cavity Squamous Cell Carcinoma
MEC	Mucoepidermoid carcinoma
Lt	Left
Rt	Right
A. poplitea	popliteal artery
ATA	anterior tibial artery
PTA	posterior tibial artery
TPT	tibioperoneal trunk
AF	fibular artery
CNN	convolutional neural network
MDCT	Multi-Detector CT
HR-pQCT	high-resolution peripheral quantitative computed tomography
iMAR	iterative metal artifact reduction
CBCT	Cone Beam CT
vBMD	volumetric bone mineral density
UHR	ultra-high-resolution
